# The effects of kisspeptin on food intake in women with overweight or obesity

**DOI:** 10.1111/dom.15086

**Published:** 2023-04-25

**Authors:** Chioma Izzi‐Engbeaya, Muhammad M. Choudhury, Bijal Patel, Beatrice Muzi, Ambreen Qayuum, Edouard G. Mills, Maheen Ahsan, Maria Phylactou, Sophie A. Clarke, Laura Aslett, Alexander N. Comninos, Ali Abbara, Tricia M. Tan, Waljit S. Dhillo

**Affiliations:** ^1^ Section of Endocrinology and Investigative Medicine, Division of Diabetes, Endocrinology and Metabolism, Department of Medicine Imperial College London London UK; ^2^ Department of Endocrinology Imperial College Healthcare NHS Trust London UK

**Keywords:** appetite control, experimental pharmacology, randomized trial, weight control

## 1 BACKGROUND

Kisspeptin is recognized to have a critical role in the control of reproductive function^1^, ^2^; however, as reproductive processes require adequate energy stores, there is increasing focus on its interaction with metabolic processes. Female (but not male) kisspeptin receptor knockout mice exhibit reduced food intake,^3^, ^4^ consistent with an orexigenic action of kisspeptin. Furthermore, some of these effects persist even after global female kisspeptin receptor knockout mice are rendered eugonadal via selective re‐expression of the kisspeptin receptor in gonadotrophin‐releasing hormone neurons.^4^ Conversely, in vitro studies show that kisspeptin stimulates anorexigenic pro‐opiomelanocortin neurons and inhibits orexigenic neuropeptide Y neurons,^5^ indicating that kisspeptin may have anorexigenic effects.

Similarly, kisspeptin administration studies in animals have yielded conflicting results. Acute central administration of kisspeptin reduces food intake in male rodents.^6^, ^7^ Acute peripheral kisspeptin administration reduces food intake in both male and female mice.^8^ However, chronic central or peripheral kisspeptin administration does not influence food intake in male rats.^9^

To date, there are only limited data investigating the effect of kisspeptin on food intake in humans. In young men with normal bodyweight, acute intravenous kisspeptin administration does not affect appetite and food intake.^10^ However, there are no studies investigating the effect of kisspeptin on food intake in women, nor in people with overweight or obesity. Therefore, we undertook this study to determine the effect of kisspeptin on appetite and food intake in women with overweight or obesity and test our hypothesis that kisspeptin would have an orexigenic effect in women with overweight or obesity.

## 2 METHODS

This single‐blinded crossover study was approved by the West London Research Ethics Committee (16/LO/0391) and registered on the ISRCTN Trial Registry (ISRCTN10114288). Written informed consent was given by all participants. Women aged 18‐60 years with a body mass index >25 kg/m^2^ were recruited via advertisements. Exclusion criteria included pregnancy, breastfeeding, psychological conditions, use of recreational or investigational drugs within the preceding 2 months, use of drugs that are known to affect appetite within the preceding 2 months, use of oral and/or transdermal oestrogen or progestin within the preceding 3 months, blood donation within 3 months of study participation, ingestion or inhalation of nicotine‐containing substances, alcoholism and malignancy.

**FIGURE 1:**
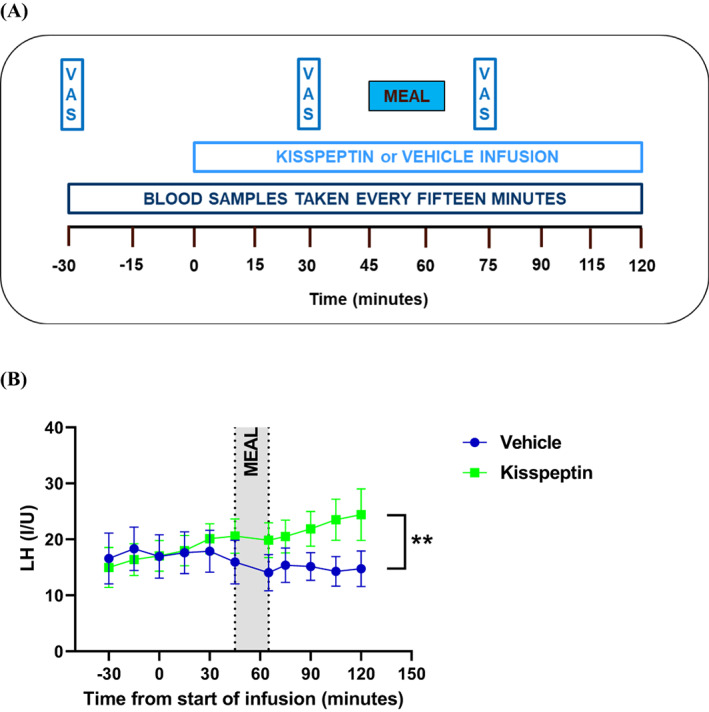
(A) After an overnight fast, participants attended the Clinical Research Unit for study visits. Blood samples were taken every 15 min from an intravenous cannula sited in one arm. Via an intravenous cannula sited in the other arm, an infusion of kisspeptin or vehicle was started at *T* = 0 min and stopped at *T* = 120 min. Visual analogue scales (VAS) for self‐rated hunger (and other parameters) were completed by participants at *T* = −30 min (pre‐infusion), *T* = 30 min (pre‐meal), and *T* = 75 min (post‐meal). Participants were given an ad libitum meal at *T* = 45 min and left to eat undisturbed until they were comfortably full. (B) Luteinizing hormone (LH) levels were higher during kisspeptin infusion compared with vehicle infusion (**p < .01).

Following a taste test to select the study meal based on Likert scores closest to ‘neither like nor dislike’, each participant attended two study visits. Each study visit was identical apart from the infusion. During one study visit, an intravenous infusion of kisspeptin‐54 at 1.0 nmol/kg/h was administered (i.e. a dose known to produce reproductive and metabolic effects in humans^10^), and rate‐matched vehicle was administered during the other study visit, with the order of infusions randomly determined using random.org. For each pre‐menopausal woman, both study visits were conducted during the same phase of the menstrual cycle. In the 24‐h period preceding each study visit, participants were asked to refrain from strenuous exercise, alcohol and caffeine. Each participant ate their evening meal at 8 pm on the night preceding each study visit and thus attended each study visit after an overnight 14‐h fast. Following a period of acclimatization, blood samples were taken regularly via an intravenous cannula (Figure 1A). At T = 0 min, the intravenous infusion was started via a second intravenous cannula in the opposite arm. The ad libitum study meal [spaghetti bolognese (1.12 kcal/g) or ricotta cannelloni (1.25 kcal/g); Waitrose], was given to participants at T = 45 min, and they were left undisturbed and instructed to eat until they were comfortably full after all watches, electronic devices and reading material were removed. Each participant received the same study meal for both of their study visits. Visual analogue scales (0‐10 cm) were completed by participants at T = −30 min, T = 30 min and T = 75 min. The infusion was stopped at T = 120 min.

Serum insulin, plasma glucose, serum luteinizing hormone (LH) and serum oestradiol were measured in the Clinical Chemistry Laboratory of Imperial College Healthcare NHS Trust. Chemiluminescent immunoassays were used to measure serum insulin (intra‐assay and inter‐assay CV: ≤7%), serum LH (intra‐assay and inter‐assay CV: ≤5%) and serum oestradiol (intra‐assay and inter‐assay CV: ≤8%). Plasma glucose was measured with a colorimetric hexokinase assay (intra‐assay and inter‐assay CV: ≤2%).

Based on our previous work, in which acute intravenous hormone infusion reduced food intake in healthy men,^11^ a sample size of 16 people would have 90% power (at a significance level of 0.05) to detect a clinically significant reduction in food intake of 2.3 ± 1.3 kcal/kg. For a woman with a weight of 100 kg, 2.3 kcal/kg would equate to 230 kcal/meal, and thus 690 kcal/day assuming three meals are eaten per day (which is equivalent to one‐third of the 2000 kcal/day recommended intake for women^12^). Statistical analysis was performed using Prism 9.4.1 (GraphPad) and data are presented as mean ± SEM. Paired t‐tests were performed on parametric data, and Wilcoxon matched‐pairs signed rank tests were performed on non‐parametric data. Two‐way ANOVAs or mixed effects models (if there were incomplete data at some timepoints) were performed on visual analogue scales, LH, oestradiol, glucose and insulin curves. Statistical significance was set at *p* < .05.

## 3 RESULTS

**FIGURE 2:**
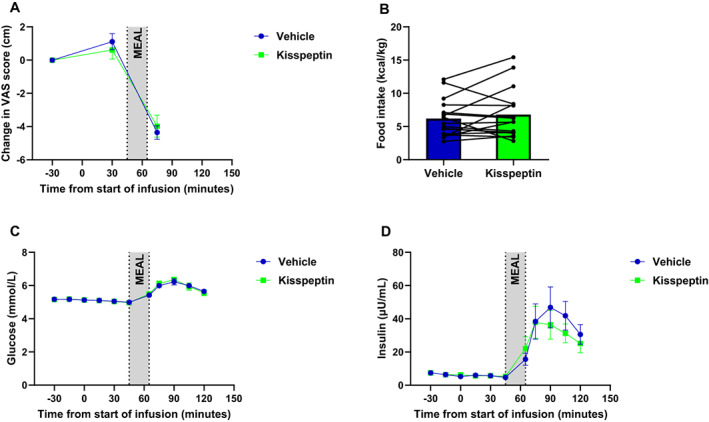
(A) There was no difference in the change from pre‐infusion hunger visual analogue scale (VAS) scores during kisspeptin infusion compared with vehicle infusion. (B) Weight‐adjusted food intake (kcal/kg = kilocalories eaten per kilogram body weight) was similar during kisspeptin and vehicle infusions. (C) Plasma glucose levels were similar during kisspeptin and vehicle infusions. (D) Serum insulin levels were similar during kisspeptin and vehicle infusions.

Seventeen women (age 49.2 ± 2.9 years, body mass index 34.3 ± 1.8 kg/m^2^, n = 5 Black, n = 12 White) were recruited and each woman completed both study visits. Baseline characteristics of the participants are summarized in Data S1, Table S1. As expected, kisspeptin infusion increased circulating LH levels (*p* < .01) (Figure 1B), confirming bioactivity of the peptide, but pre‐meal oestradiol levels (*p* = .34) were similar during kisspeptin and vehicle infusions. Compared with vehicle infusion, kisspeptin infusion did not affect self‐rated hunger (*p* = .41) (Figure 2A and Data S1, Figure S1A, B), self‐rated fullness (Data S1, Figure S1C, D) and self‐rated eating pleasure (Data S1, Figure S1E, F). Furthermore, kisspeptin infusion did not affect food intake (vehicle 6.2 ± 0.6 kcal/kg vs. kisspeptin 6.8 ± 0.9 kcal/kg, *p* = .33) (Figure 2B). In addition, kisspeptin administration did not affect glucose (*p* = .97) (Figure 2C) or insulin (*p* = .68) (Figure 2D) levels. No side effects were reported by the participants, and both heart rate and blood pressure were unaffected by kisspeptin infusion (Data S1, Figure S2).

## 4 CONCLUSIONS

In this study, we showed that a biologically active dose of kisspeptin did not affect self‐reported appetite and did not affect objectively measured food intake in women with overweight or obesity. In addition, intravenous administration of kisspeptin did not influence preprandial and postprandial glucose and insulin levels in women with overweight or obesity. This is the first study to report on the effect of kisspeptin on appetite, food intake, glucose and insulin levels in women, and in people who are overweight or have obesity. These results are consistent with a study of kisspeptin administration in men with normal bodyweight.^10^ Furthermore, as previously reported in men,^10^ kisspeptin did not enhance glucose‐stimulated insulin secretion when glucose levels remained within physiological limits in women. Kisspeptin administration robustly increased LH levels, in keeping with its known reproductive effects in women with normal weight.^2^ Oestradiol is known to influence appetite,^13^ but oestradiol levels remained unchanged before consumption of the meal.

Because of the contradictory preclinical literature,^6^, ^7^, ^8^, ^9^ it is necessary to conduct studies in humans to determine if there are species‐specific effects. Furthermore, as sexually dimorphic effects on food intake in kisspeptin receptor knockout rodents have been reported,^3^ this study enhances our knowledge of the metabolic effects of kisspeptin in humans across genders. As the therapeutic potential of kisspeptin for use in metabolic disorders is being investigated,^14^ this study provides reassurance that kisspeptin‐based treatments are unlikely to be directly orexigenic in women and in people with overweight or obesity.

The strengths of this study include the crossover design, which minimized inter‐individual variation that may confound results. Furthermore, the evidence of other known biological effects of kisspeptin (i.e. increase in LH) provides confidence that the absence of the effect of kisspeptin on food intake was not due to the study being underpowered or the use of an inadequate dose of kisspeptin. Limitations of this study include the short duration of kisspeptin administration and insufficient power to detect the effects of kisspeptin in subgroups of study participants.

On the background of conflicting animal data and the absence of published data in women, this study provides important data on women that can inform the ongoing development of kisspeptin‐based treatments. Future work involving chronic kisspeptin administration will be helpful in elucidating the metabolic effects of kisspeptin in humans.

## AUTHOR CONTRIBUTIONS

Conceptualization was performed by CI‐E, TT and WSD; the investigation was performed by CI‐E, MC, BP, BM, AQ, EM, MA, MP, SC, LA, ANC and AA; CI‐E and MC analysed the data; writing of the original draft was performed by CI‐E, while writing of the review and editing was performed by all the authors.

## CONFLICTS OF INTEREST STATEMENT

The authors have no conflicts of interest to declare.

### PEER REVIEW

The peer review history for this article is available at https://www.webofscience.com/api/gateway/wos/peer-review/10.1111/dom.15086.

## Supporting information


**Data S1:** Supporting Information

## Data Availability

The data that support the findings of this study are available from the corresponding author upon reasonable request.

## References

[1] Seminara SB , Messager S , Chatzidaki EE , et al. The GPR54 gene as a regulator of puberty. N Engl J Med. 2003;349:1614‐1627. 14573733 10.1056/NEJMoa035322

[2] Dhillo WS , Chaudhri OB , Thompson EL , et al. Kisspeptin‐54 stimulates gonadotropin release most potently during the preovulatory phase of the menstrual cycle in women. J Clin Endocrinol Metab. 2007;92:3958‐3966. 17635940 10.1210/jc.2007-1116

[3] Tolson KP , Garcia C , Yen S , et al. Impaired kisspeptin signaling decreases metabolism and promotes glucose intolerance and obesity. J Clin Invest. 2014;124:3075‐3079. 24937427 10.1172/JCI71075 PMC4071390

[4] Velasco I , Leon S , Barroso A , et al. Gonadal hormone‐dependent vs. ‐independent effects of kisspeptin signaling in the control of body weight and metabolic homeostasis. Metabolism. 2019;98:84‐94. 31226351 10.1016/j.metabol.2019.06.007

[5] Fu LY , van den Pol AN . Kisspeptin directly excites anorexigenic proopiomelanocortin neurons but inhibits orexigenic neuropeptide Y cells by an indirect synaptic mechanism. J Neurosci. 2010;30:10205‐10219. 20668204 10.1523/JNEUROSCI.2098-10.2010 PMC2933146

[6] Stengel A , Wang L , Goebel‐Stengel M , Taché Y . Centrally injected kisspeptin reduces food intake by increasing meal intervals in mice. Neuroreport. 2011;22:253‐257. 21386700 10.1097/WNR.0b013e32834558df PMC3063509

[7] Saito R , Tanaka K , Nishimura H , et al. Centrally administered kisspeptin suppresses feeding via nesfatin‐1 and oxytocin in male rats. Peptides. 2019;112:114‐124. 30562556 10.1016/j.peptides.2018.12.003

[8] Dong TS , Vu JP , Oh S , Sanford D , Pisegna JR , Germano P . Intraperitoneal treatment of kisspeptin suppresses appetite and energy expenditure and alters gastrointestinal hormones in mice. Dig Dis Sci. 2020;65:2254‐2263. 31729619 10.1007/s10620-019-05950-7

[9] Castellano JM , Navarro VM , Fernandez‐Fernandez R , et al. Changes in hypothalamic KiSS‐1 system and restoration of pubertal activation of the reproductive axis by kisspeptin in undernutrition. Endocrinology. 2005;146:3917‐3925. 15932928 10.1210/en.2005-0337

[10] Izzi‐Engbeaya C , Comninos AN , Clarke SA , et al. The effects of kisspeptin on beta‐cell function, serum metabolites and appetite in humans. Diabetes Obes Metab. 2018;20:2800‐2810. 29974637 10.1111/dom.13460 PMC6282711

[11] Izzi‐Engbeaya C , Jones S , Crustna Y , et al. Effects of glucagon‐like peptide‐1 on the reproductive axis in healthy men. J Clin Endocrinol Metab. 2020;105:1119‐1125. 32052032 10.1210/clinem/dgaa072 PMC7082082

[12] Public Health England . Government Dietary Recommendations. 2016 https://assets.publishing.service.gov.uk/government/uploads/system/uploads/attachment_data/file/618167/government_dietary_recommendations.pdf Accessed on March 21, 2023.

[13] Dragano N , Milbank E , Lopez M . Estradiol and appetite: to eat or not to eat. Mol Metab. 2020;42:101061. 32771697 10.1016/j.molmet.2020.101061 PMC7484542

[14] Guzman S , Dragan M , Kwon H , et al. Targeting hepatic kisspeptin receptor ameliorates nonalcoholic fatty liver disease in a mouse model. J Clin Invest. 2022;132:e145889. 35349482 10.1172/JCI145889 PMC9106350

