# Supplementary material for: The effects of kisspeptin on food intake in women with overweight or obesity
Source: Diabetes Obes Metab. 2023 Apr 25;25(8):2393–7. doi: 10.1111/dom.15086 (PMC10946989; doi:10.1111/dom.15086)
Supplement: Supplementary file 1 — Data S1: Supporting Information [file DOM-25-2393-s001.docx]

**Supplementary Table 1 – Baseline characteristics of study participants**

| **Participant** | **Age (years)** | **Body Mass Index (kg/m^2^)** | **Ethnic background** | **Menopausal Status** | **Co-Morbidities** | **Medications** |
| --- | --- | --- | --- | --- | --- | --- |
| 1 | 37 | 45.3 | Black | Pre-menopausal | Pre-diabetes | Mirena® IUS |
| 2 | 45 | 27.0 | White | Pre-menopausal | None | None |
| 3 | 21 | 44.8 | Black | Pre-menopausal | None | None |
| 4 | 59 | 25.9 | Black | Post-menopausal | Hypertension | Ramipril |
| 5 | 53 | 25.5 | White | Post-menopausal | None | None |
| 6 | 57 | 26.5 | White | Post-menopausal | None | None |
| 7 | 54 | 45.6 | White | Post-menopausal | Pre-diabetes, Hypertension, Hypothyroidism | Amlodipine, Levothyroxine |
| 8 | 54 | 39.3 | White | Post-menopausal | None | None |
| 9 | 57 | 28.4 | White | Post-menopausal | Obstructive Sleep Apnoea | None |
| 10 | 54 | 29.2 | White | Post-menopausal | None | None |
| 11 | 51 | 42.7 | White | Pre-menopausal | None | Mirena® IUS |
| 12 | 61 | 33.1 | White | Post-menopausal | None | None |
| 13 | 50 | 30.4 | Black | Pre-menopausal | None | None |
| 14 | 49 | 36.8 | Black | Pre-menopausal | None | None |
| 15 | 50 | 39.1 | White | Post-menopausal | NAFLD | None |
| 16 | 61 | 26.4 | White | Post-menopausal | None | None |
| 17 | 23 | 37.5 | White | Pre-menopausal | None | None |

Demographic, anthropometric and clinical characteristics of the study participants. IUS – intrauterine system. NAFLD – non-alcoholic fatty liver disease.

**Supplementary Figure S1**

**Supplementary Figure S2**

**Supplementary Figure Legends**

**Figure S1**

Figure S1A: Mean self-rated hunger visual analog scale (VAS) scores during vehicle infusion and kisspeptin infusion were similar 30 minutes after the start of each infusion, prior to ingestion of the *ad libitum* study meal (vehicle 5.9±0.5cm vs kisspeptin 5.2±0.7cm, p=0.33).

Figure S1B: Mean self-rated hunger visual analog scale (VAS) scores during vehicle infusion and kisspeptin infusion were similar 75 minutes after the start of each infusion, following ingestion of the *ad libitum* study meal (vehicle 0.4±0.2cm vs kisspeptin 0.5±0.2cm, p=0.87).

Figure S1C: Mean self-rated fullness visual analog scale (VAS) scores during vehicle infusion and kisspeptin infusion were not significantly different 30 minutes after the start of each infusion, prior to ingestion of the *ad libitum* study meal (vehicle 1.9±0.5cm vs kisspeptin 2.1±0.5cm, p=0.41).

Figure S1D: Mean self-rated fullness visual analog scale (VAS) scores during vehicle infusion and kisspeptin infusion were not significantly different 75 minutes after the start of each infusion, following ingestion of the *ad libitum* study meal (vehicle 7.7±0.5cm vs kisspeptin 7.7±0.5cm, p=0.87).

Figure S1E: Mean self-rated visual analog scale (VAS) scores of ‘how pleasant it would be to eat’ during vehicle infusion and kisspeptin infusion were similar 30 minutes after the start of each infusion, prior to ingestion of the *ad libitum* study meal (vehicle 6.3±0.6cm vs kisspeptin 6.3±0.6cm, p=0.95).

Figure S1F: Mean self-rated visual analog scale (VAS) scores of ‘how pleasant it would be to eat’ during vehicle infusion and kisspeptin infusion were not significantly different 75 minutes after the start of each infusion, following ingestion of the *ad libitum* study meal (vehicle 1.0±0.2cm vs kisspeptin 0.6±0.3cm, p=0.19).

**Figure S2**

Figure S2A: Compared to vehicle infusion, kisspeptin infusion did not have a significant effect on the heart rate of the participants (p=0.83). bpm – beats per minute.

Figure S2B: Compared to vehicle infusion, kisspeptin infusion did not have a significant effect on the systolic blood pressure of the participants (p=0.67). mmHg – millimetres of mercury.

Figure S2C: Compared to vehicle infusion, kisspeptin infusion did not have a significant effect on the diastolic blood pressure of the participants (p=0.65). mmHg – millimetres of mercury.
